# Quasi-Isotropic and Pseudo-Ductile Highly Aligned Discontinuous Fibre Composites Manufactured with the HiPerDiF (High Performance Discontinuous Fibre) Technology

**DOI:** 10.3390/ma12111794

**Published:** 2019-06-03

**Authors:** M. L. Longana, H. Yu, J. Lee, T. R. Pozegic, S. Huntley, T. Rendall, K. D. Potter, I. Hamerton

**Affiliations:** 1 Bristol Composites Institute (ACCIS), Department of Aerospace Engineering, School of Civil, Aerospace, and Mechanical Engineering, Queen’s Building, University of Bristol, University Walk, Bristol BS8 1TR, UK; H.Yu@bath.ac.uk (H.Y.); juhyeong.lee@bristol.ac.uk (J.L.); t.pozegic@bristol.ac.uk (T.R.P.); samantha.huntley@bristol.ac.uk (S.H.); thomas.rendall@bristol.ac.uk (T.R.); k.potter@bristol.ac.uk (K.D.P.); ian.hamerton@bristol.ac.uk (I.H.); 2 Department of Mechanical Engineering, University of Bath, Bath BA2 7AY, UK

**Keywords:** aligned discontinuous fibre composites, quasi-isotropic laminate, pseudo-ductility

## Abstract

Conventional composite materials reinforced with continuous fibres display high specific strength but have a number of drawbacks including: the elastic-brittle behaviour, difficulties in producing defect-free components of complex shape with high-volume automated manufacturing processes, and inherent lack of recyclability. Highly aligned, discontinuous fibre-reinforced composites (ADFRCs) are truly beneficial for mass production applications, with the potential to offer better formability and comparable mechanical properties with continuous fibre-reinforced composites. In previous publications, the High Performance Discontinuous Fibre (HiPerDiF) technology has been shown to offer the possibility to intimately hybridise different types of fibres, to achieve pseudo-ductile tensile behaviour, and remanufacture reclaimed fibres into high-performance recycled composites. However, to date, the work has been conducted with unidirectional (UD) laminates, which is of limited interest in engineering applications with mechanical stresses acting across many directions; this paper reports, for the first time, the mechanical behaviour of quasi-isotropic (QI) ADFRCs. When compared with randomly-oriented discontinuous fibre composites (RODFRCs), QI ADFRCs offer enhanced stiffness (+26%) and strength (+77%) with higher consistency, i.e., a reduction of the coefficient of variance from the 25% of RODFRCs to the 6% of ADFRCs. Furthermore, hybrid QI ADFRCs retain the pseudo-ductility tensile behaviour previously observed in unidirectional (UD) lay-up.

## 1. Introduction

Continuous fibre-reinforced composites achieve high structural performance with minimal weight, replacing metals in many traditional primary and secondary applications in the aerospace sector, but increasingly in wind turbines, automotive structures, and gas and oil pipes [1]. However, the long and continuous nature of the fibre reinforcement and the tendency to fail catastrophically, limits design flexibility. Furthermore, continuous fibres prepregs cannot follow the geometry of complex mould shapes and deposition paths, generating manufacturing defects such as in-plane/out-of-plane wrinkles and resin-rich pockets, which significantly reduce the mechanical properties of composite parts [2].

Highly-Aligned Discontinuous Fibre Reinforced Composites (ADFRCs) have the potential to offer mechanical properties (stiffness, strength, and failure strain) comparable with those of continuous fibre composites provided that the fibre aspect ratio is sufficiently high to allow load transfer and attain fibre failure instead of pull-out (fibre/matrix interface failure) [3]. Moreover, ADFRCs make it possible to overcome three of the key limitations of conventional continuous fibre composite materials:

- the lack of ductility, i.e., their substantially elastic-brittle behaviour, by intimately hybridising different types of fibres or exploiting pull-out mechanisms [4,5,6,7,8,9];
- the difficulties in producing defect-free components of complex shape with high-volume automated manufacturing processes [10,11,12];
- the issues associated with implementing a sustainable material lifecycle, e.g., the integration of production and end-of-life recycled waste in a circular economy model, through the remanufacturing of discontinuous reclaimed fibres into high-performance recycled composite materials [13,14,15,16,17,18,19].

**Figure 1:**
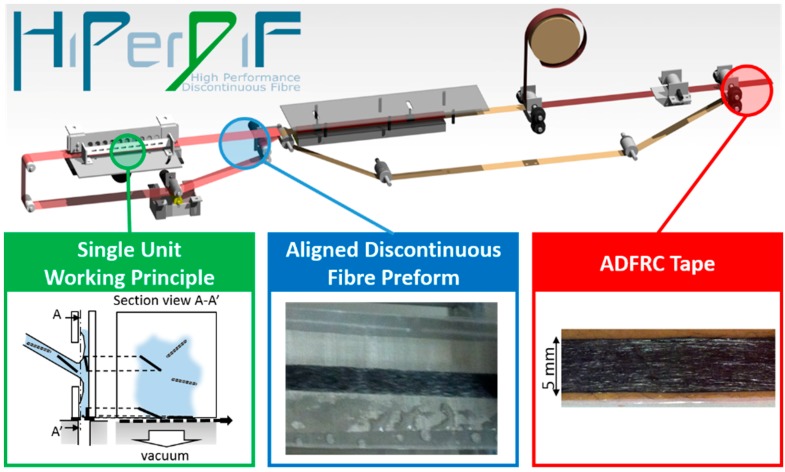
The HiPerDiF fibre alignment machine.

Overviews of discontinuous fibre alignment techniques have been provided by Sunny et al. [20] and Such et al. Some of these techniques are derived from paper-making techniques and use a high viscosity suspension medium [21]. The High Performance Discontinuous Fibre (HiPerDiF) method, shown in Figure 1, invented at the University of Bristol (UoB), is a new and effective way to manufacture sustainable high-performance ADFRCs and potentially delivers the route to high throughput [3]. HiPerDiF has been used to remanufacture reclaimed fibres into high-performance, recycled carbon fibre composites [13,16] to produce closed loop recyclable materials [14,15] and functionalised composites from sustainable sources [17]. Most importantly, the HiPerDiF technology allows the intimate mixing of different types of synthetic fibres to achieve a pseudo-ductile behaviour, i.e., a plateau in the stress-strain curve, with intermingled [4], intraply [5], interlaminated [6,13], and hierarchical hybrids [6]. In all the aforementioned architectures, pseudo-ductility is achieved through a mechanism comparable to the one described by Jalalvand et al. [22]; the stable and progressive fragmentation of low strain-to-failure fibres allows load transfer on to the higher strain-to-failure fibres without the occurrence of global material failure or catastrophic delamination. This mechanism has been exploited with continuous fibre thin-ply laminates laid-up in unidirectional (UD) [23] and quasi-isotropic (QI) architectures [24]; however, the work conducted thus far to investigate the behaviour of ADFRCs has only been performed on UD laminates and in most engineering applications it is usually necessary to balance the load-carrying capability in multiple directions.

The work conducted herein aims to investigate the behaviour of ADFRCs laminates in a QI lay-up. In the first instance, the QI ADFRCs are compared with randomly-oriented discontinuous fibre composites (RODFRCs) [25]. Secondly, the possibility of retaining a pseudo-ductile behaviour in a QI lay-up for the hybrid ADFRCs that demonstrated functional characteristics in a UD laminate is also investigated [26].

## 2. Materials & Manufacturing

The ADFRCs used in this study were manufactured with the HiPerDiF (High Performance Discontinuous Fibre) technology [3]. The unique fibre orientation mechanism of the HiPerDiF technology, realised at lab-scale with the machine shown by Figure 1, can be described as follows, with further information found in Ref: [3].

A suspension of fibres dispersed in water, delivered by a peristaltic pump, is sprayed through a nozzle between two parallel plates kept at a defined distance. Provided that the fibre length is less than the plates’ separation distance, the sudden momentum change generated by the impact of the fibres against the furthermost plate aligns them parallel to the plate itself. The fibres then fall on to a conveyor mesh belt where the alignment is finalised and most of the water is removed via a vacuum suction system placed underneath the mesh belt. The aligned fibre preform is transferred to a second conveyer belt, made with a polytetrafluoroethene (PTFE)-coated glass fibre cloth, that transports it under an infrared heater where the drying is completed. At this point, it is possible to couple the dry fibre preform with a resin film and create a partially impregnated tape, i.e., an ADF prepreg, by applying heat and pressure.

### 2.1. Materials

The HiPerDiF machine can process a wide variety of synthetic, virgin and reclaimed, and natural fibres (and has previously been used to align different forms of carbon, glass, Kevlar, viscose, poly(vinyl alcohol), cellulose and flax). In previous research [3,4,5,6], it was found that by combining fibres displaying high mechanical strength (PAN-based carbon) or high modulus (pitch-based carbon) or greater elongation at break (E-glass), it was possible to produce thin ADFRC laminate tapes with pseudo-ductile behaviour. Consequently, high tensile strength carbon fibres (HSC, C124, TohoTENAX, Tokyo, Japan), high tensile modulus carbon fibres (HMC, Granoc XN-90, NGF, Himeji, Japan), and E-glass fibres (EG, C100, Vetrotex, Wallingford, UK) were also used for this study, see Table 1 for selected properties. The fibres were coupled with a 43 gsm areal weight K-51 resin film (SKChemicals, Gyeonggi-do, Republic of Korea), a bisphenol-based toughened epoxy resin with a density of 1.21 g/cm^3^.

### 2.2. Specimen Manufacturing

Three specimen formats used in this research work were manufactured as described below.

**Randomly-oriented discontinuous fibre composites (RODFRC).** Three millimetre fibres were dispersed in a water bath (*ca.* 100 mg/L) under stirring with a stainless-steel mesh placed at the bottom. The fibres were left to deposit on the mesh, which was then slowly lifted to sieve out the water. The resulting mat of fibres, still resting on the stainless-steel mesh, were then dried in an oven at 90 °C for 60 min. The K-51 resin film was deposited on top of the fibre mat; partial impregnation was achieved by applying heat (60 °C) and moderate pressure through a hand-held roller. The obtained semi-prepreg mat was cut into 5 mm × 150 mm strips; four strips were laid-up in a semi-closed mould to obtain the RODFRC tensile test specimens; the curing cycle is described below.

**Figure 2:**
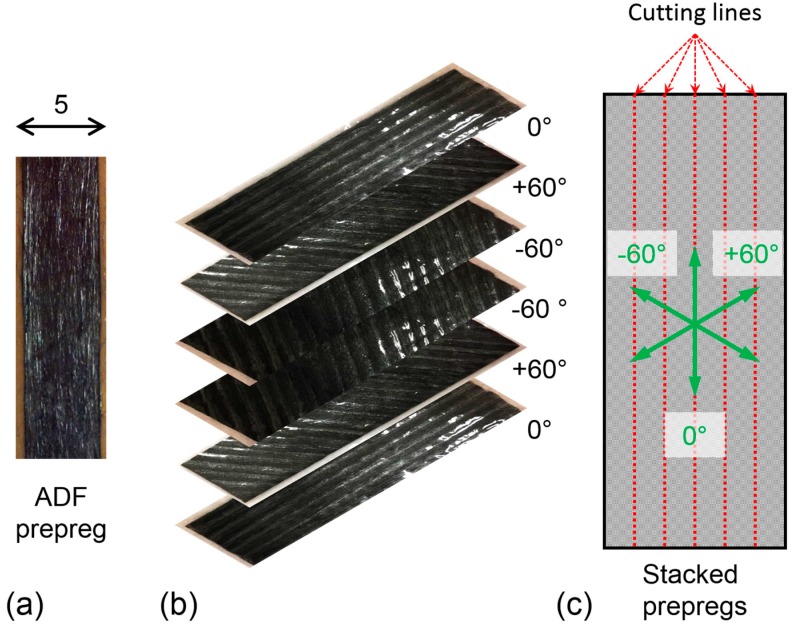
Images showing QI Specimen preparation: (**a**) Aligned discontinuous fibres prepreg tape, (**b**) lay-up and stacking sequence of ADFRC tapes, (**c**) Specimen cutting lines on uncured ADFRC stacked tapes.

**Unidirectional (UD) ADFRC specimens.** To obtain the UD ADFRC tensile test specimens, four layers of the ADF prepreg tape, depicted in Figure 2a, were laid-up in a semi-closed mould and cured as described below.

**Quasi-Isotropic (QI) ADFRC specimens.** The HiPerDiF machine produces ADF prepreg of 5 mm width, and several tapes were placed by hand alongside one another to form a 40 mm × 150 mm ply. Subsequent plies were added to obtain the final lay-up, as shown in Figure 2b; the QI lay-up was achieved by stacking the ADF prepregs according to the [0/+60/−60]s sequence. The stacked prepregs were then cut into 5-mm-wide strips, as shown in Figure 2c, and placed in a semi-closed steel mould. All the specimens were cured following the same procedure and curing cycle: the semi-closed mould was placed in a vacuum bag and cured in autoclave for 135 min at a temperature of at 135 °C and a pressure of 6 bar. After removing the specimens from the mould, burrs were gently removed from all edges using sand paper. Glass fibre/epoxy end-tabs with a length of 50 mm were attached using epoxy adhesives (Aradite 2014, Huntsman, The Woodlands, Texas, USA) to all the specimens leaving a 50-mm-long gauge length.

## 3. Testing Methodology

Tensile tests were performed at a 1 mm/min cross-head displacement speed on an electro-mechanical testing machine equipped with a 10 kN load cell (Shimadzu, Kyoto, Japan) according to ASTM [27]. The strain was measured with a video extensometer (IMETRUM, Bristol, UK): white speckles were spray-painted over a black background allowing for a 42.5 mm ± 2.5 mm gauge length.

The ignition loss method [28] was applied to measure the fibre volume fraction (V_f_) of the RODFRC and ADFRC specimens. The epoxy resin was burned off by placing the samples in a furnace at a temperature of 500 °C for 150 min. The samples were weighed before and after the resin burn off with a resolution of 0.1 mg. The V_f_ was calculated based on the sample weights and the densities of the fibres and cured epoxy resin.

## 4. Experimental Results & Discussion

### 4.1. 100%. HSC Fibre Specimens

**Figure 3:**
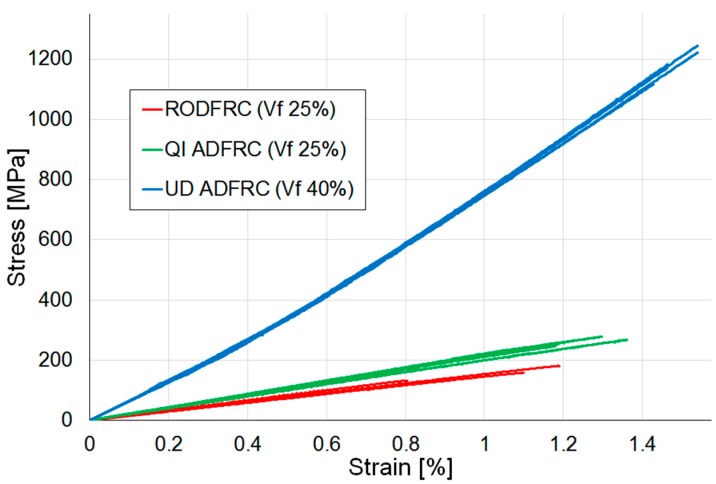
Tensile stress-stain curves for 100% HSC discontinuous fibre composites.

A first round of tests was performed on three sets of five specimens manufactured using 100% HSC fibres to compare the mechanical performances of RODFRC, UD ADFRC, and QI ADFRC. The V_f_ values were measured to be 25 ± 2% for the RODFRC and QI ADFRC, and 40 ± 2% for the UD ADFRC [6]. The stress-strain curves obtained for the laminates are shown in Figure 3.

**Figure 4:**
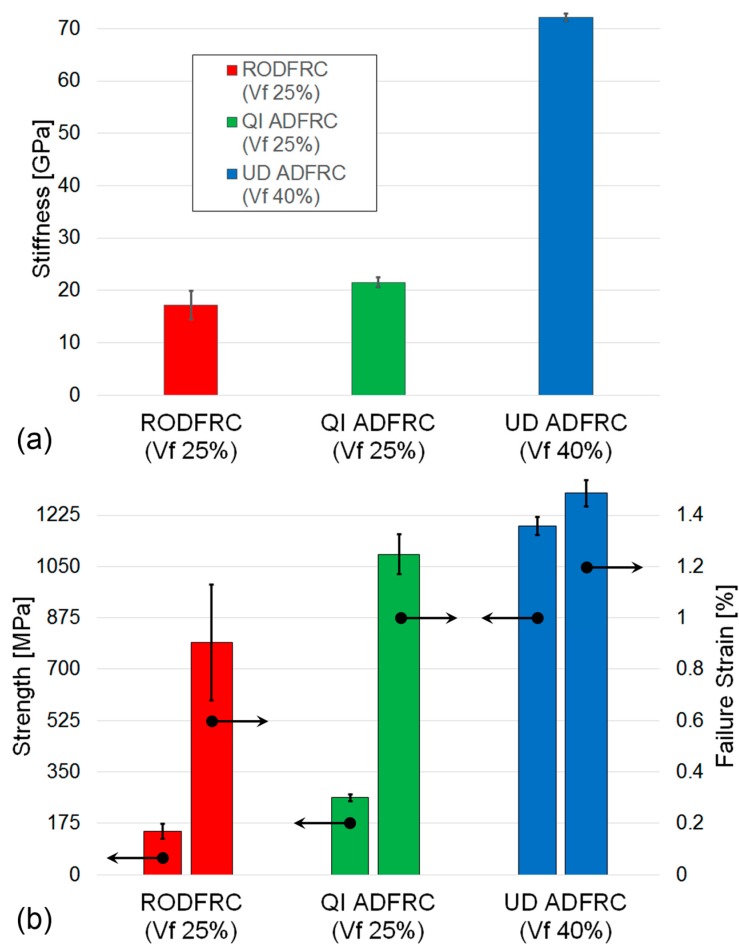
100% HSC discontinuous fibre composites tensile test results: (**a**) stiffness, (**b**) failure properties.

The calculated material properties are summarized in Table 2 and shown in Figure 4: The stiffness is identified as the gradient of the stress-strain curve between 0.1% and 0.3% strain [27]. Strength and failure strain are measured values corresponding to the specimens’ loss of integrity and are visible as a sudden drop in the stress-strain curve displayed by the machine during testing and loss of any material connection between the top and bottom end-tabbed parts of the specimen.

**Figure 5:**
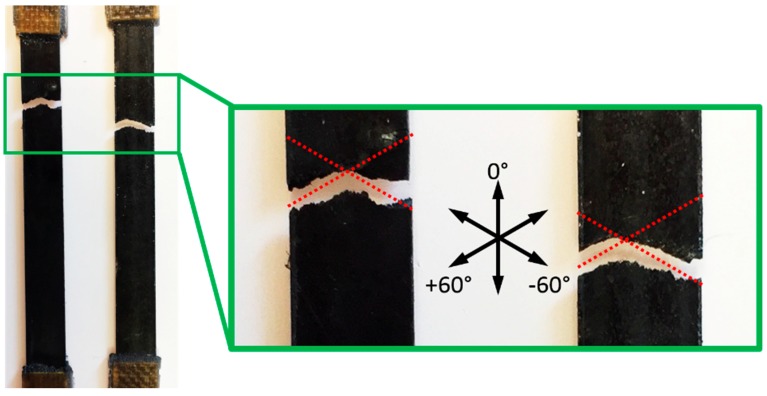
Failed QI ADFRC specimens.

As expected, the UD ADFRC offers the best tensile mechanical properties. However, it is more relevant to notice how these are higher for the QI ADFRC than for the RODFRC even though the fibre length and volume fraction are the same. Specifically, tensile modulus, strength, and strain to failure increased by 26%, 77%, and 38% respectively. Moreover, the QI ADFRC specimens showed more consistent results than those recorded for RODFRCs, with coefficients of variation in the range of ±6% for the former versus 15–25% for the latter. While the ROFRC specimens inevitably contain randomly-distributed, entangled fibre clusters, resin pockets and voids that are likely to cause reductions in mechanical properties; QI-ADFRCs offer more organised micro-structures. This is also reflected in the specimen fracture section caused by a tensile load (Figure 5): both the QI continuous fibre-reinforced composites and the QI ADFRC specimens failed along the ±60° direction. Analysis of the fractured surfaces confirms that matrix cracks in the ±60° layers between the fibres propagate through the thickness direction causing the failure of the whole specimen [29].

**Figure 6:**
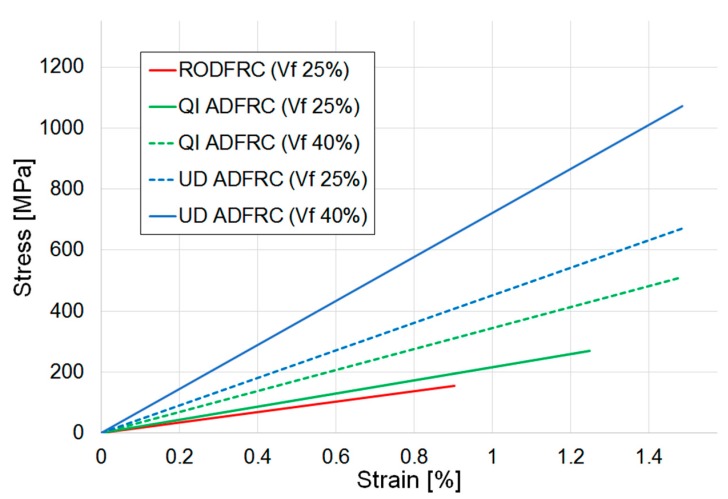
Comparison of representative tensile stress-stain curves for 100% HSC discontinuous fibre composites normalised to common V_f_ values.

In common with conventional composites, where mechanical properties are heavily dependent on the quantity of the reinforcing fibre present, higher QI ADFRC properties could be achieved with an increase in fibre volume fraction. The QI ADFRC samples were produced with 25% V_f_ in this work and, in order to make a valid comparison with the UD AFRC, normalisation was required [6]. Once the samples have been normalised to a common value of 40% V_f_, then it is suggests that values of stiffness and strength of 35 GPa and 520 MPa respectively are obtainable for an QI ADFRC (40% V_f_), as shown in Figure 6.

This is much higher than other short fibre composites: For instance, injection-moulded PA6 or poly(acrylonitrile-butadiene-styrene) (ABS) composites reinforced with short carbon fibres yield tensile moduli in the range of 5–12 GPa [30,31]; this is also comparable with the stiffness recorded for 50-mm chopped-stand randomly-oriented mat composites [32]. Previously, the HiPerDiF method has been successfully used to produce a small laminate with a V_f_ of 55% [3]: suggesting the potential for further improvements in mechanical properties.

### 4.2. Pseudo-Ductile ADFRCs

**Figure 7:**
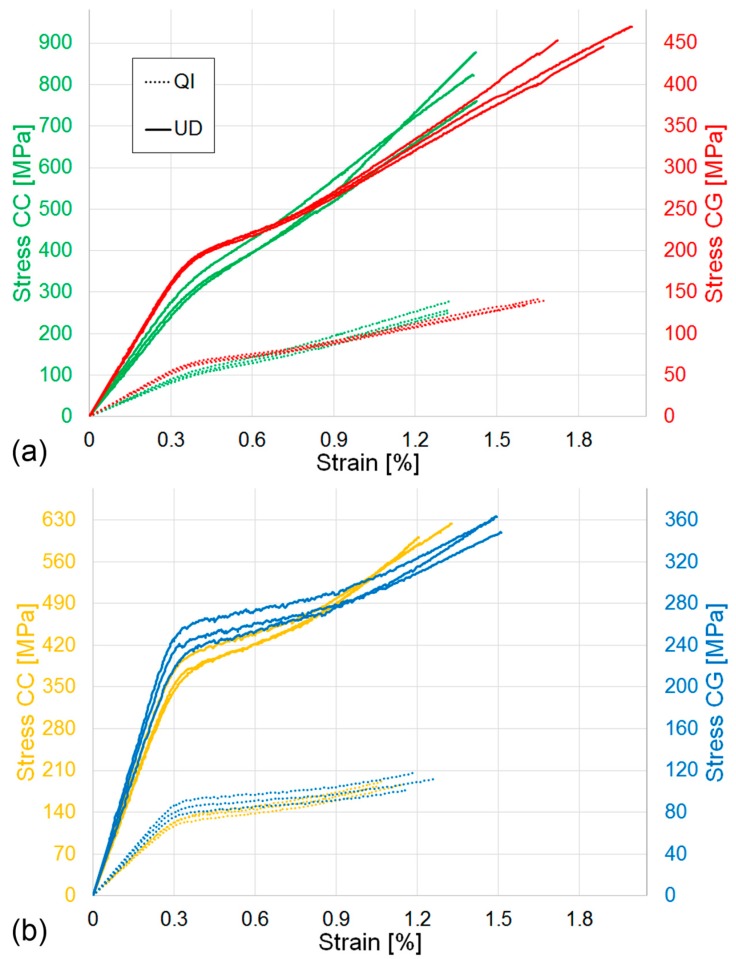
Tensile stress-stain curves for pseudo-ductile ADFRCs: (**a**) 20%HMC/80%HSC (CC) and 20%HMC/80%EG (CG); (**b**) 50%HMC/50%HSC (CC) and 40%HMC/60%EG (CG).

This second round of tests was performed on eight sets of three specimens each and the compositions were based on previously reported results [6]. Consequently, four combinations were chosen to exemplify (a) low HMC content and high (HSC and EG) content (e.g., 20%HMC/80%HSC and 20%HMC/80%EG) and (b) high HMC and high (HSC and EG) content (e.g., 50%HMC/50%HSC and 40%HMC/60%EG). These were selected as the compositions in (a) should allow higher yield strains to be obtained, while the compositions in (b) should allow higher pseudo-ductile strains to be obtained. For each of the selected combinations, both UD and QI lay-ups were considered. An average V_f_ of 37 ± 1% was measured across the manufactured sets for the UD and 28 ± 1% for the QI specimens; the difference can be explained by considering that the UD allowed a higher amount of excess resin to escape from the semi-closed mould during curing. The stress-strain curves obtained for the four combinations are shown in Figure 7 and it is clear that the pseudo-ductility previously observed in small UD laminates is also preserved in the QI lay-up.

**Figure 8:**
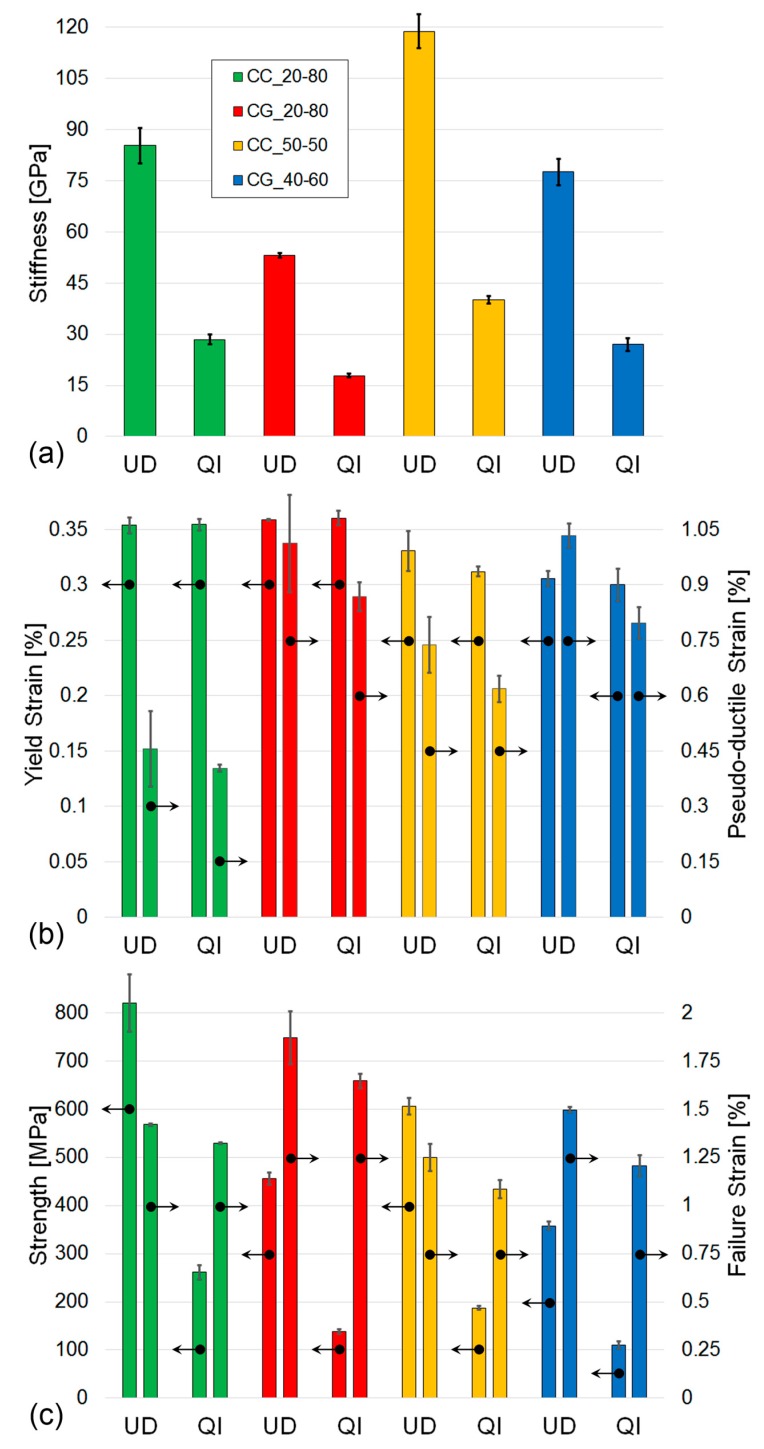
Pseudo-ductile ADFRCs tensile test results: (**a**) stiffness, (**b**) pseudo-ductile parameters, and (**c**) failure properties.

This can be explained by considering that the failure strain of the HMC fibres is ≈0.4% while, in the 100% HSC QI specimens, the matrix cracks appeared in the ±60° layers at a strain of ≈1.25%. These observations are particularly relevant as this outcome will give designers the possibility to exploit both the manufacturing benefits of ADFRCs and the functional properties of intermingled hybrids. The calculated material properties are summarised in Table 3 and shown in Figure 8.

**Figure 9:**
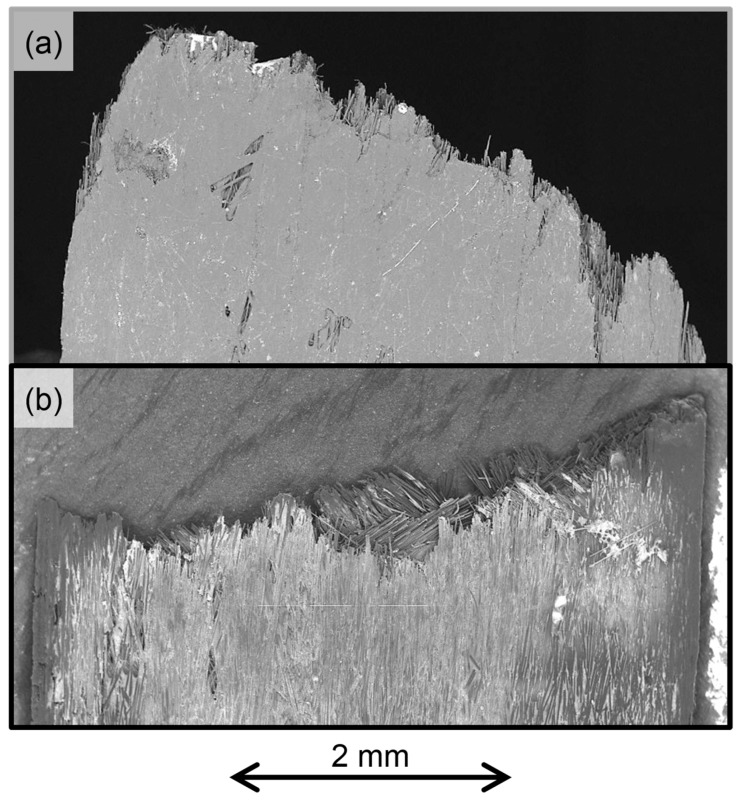
Failure surface SEM images: (**a**) UD; (**b**) QI.

The stiffness is identified as the gradient in the stress-strain curve between 0.1% and 0.3% strain [27], and the yield strain is determined from the intersection of the two linear regions [6], i.e., the linear-elastic region and the fragmentation plateau, which is equivalent to the definition of transition strain described in the ASTM standard to identify the tensile properties of the polymeric matrix composites [27]. The pseudo-ductile strain is the difference between the failure strain and the elastic strain at the same stress calculated using the initial stiffness [4], while the strength and failure strain are the values of stress and strain corresponding to the specimens’ loss of integrity. Overall, the results are in line with those obtained previously [4,6] and is common to both UD and QI laminates; i.e., an increase in the HMC content of the laminate leads to an increase in initial stiffness, however, it slightly anticipates the yield and failure strain. The effect of the lay-up sequence on the pseudo-ductile properties is particularly interesting. As expected, considering the effects of both lamination sequence and lower V_f_, the stiffness is reduced when transitioning from UD to QI, but the yield strain remains substantially unchanged by the different lay-ups, remaining predominantly driven by the HMC fibres fragmentation, as explained above. The QI failure strain is reduced by about 13 ± 5% when compared with the UD specimens, this translates to a reduction of 16 ± 5% of the pseudo ductile strain. In the SEM images of the UD and QI pseudo-ductile ADFRCs, as shown in Figure 9, the differences in the failure shapes, which are already shown in Figure 5, appear more evident.

## 5. Conclusions

The mechanical behaviour of quasi-isotropic aligned discontinuous fibre composites has been analysed in this paper. To investigate the effects of the fibre architecture on the material mechanical performances, both QI ADFRCs and RODFRCs have been compared. The more organised micro-structure of QI ADFRCs allows superior tensile modulus (+26%), strength (+77%), and strain to failure (+38%) with a higher consistency, with coefficients of variation in the range of ±6% *versus* ±15–25% for the RODFRCs. Importantly, by increasing the fibre content to the levels already achieved for conventional laminates (V_f_ = 40–55%), it will be possible to reach even better properties.

To investigate the possibility to retain functional properties, i.e., pseudo-ductility, in a QI lay-up, four different hybrid materials have been manufactured and tested using high modulus carbon as low elongation fibres and high strength or e-glass as high elongation fibres. The fragmentation of the low elongation fibres is triggered at a lower strain than the overall QI failure strain and this mechanism is responsible for the pseudo-ductile tensile behaviour observed in the ADFRCs. Furthermore, the yield point remains unaltered from the UD configuration, while the failure strain, and consequently the pseudo-ductile strain, are slightly reduced. Work is currently underway to scale up the HiPerDiF machine to increase both the throughput of fibre processed and the V_f_ that can be achieved, resulting in larger ADFRC laminates and the possibility to extend the ADFRC characterisation campaign to a wide variety of loading conditions, e.g., compression, bending, fatigue and high strain rate as well as material behaviour in the vicinity of notches represented by open holes and combinations of a wider range of synthetic, reclaimed and natural fibres.

## Figures and Tables

**Table 1:** Fibre Properties [4].

		C124, TohoTENAX (HSC)	Granoc XN-90, NGF (HMC)	C100, Vetrotex (EG)
Material		Polyacrylontrile. based carbon	Pitch based carbon	E-glass
Diameter	[μm]	7	10	7
Length	[mm]	3	3	3
Density	[g/cm^3^]	1.82	2.21	2.60
Stiffness	[GPa]	225	860	73
Strength	[MPa]	4344	3430	2400
Failure Strain	[%]	1.93	0.398	3.29

**Table 2:** 100% HSC tensile test results. (CV = Coefficient of variation).

		RODFRC (V_f_ 25%)	QI ADFRC (V_f_ 25%)	UD ADFRC (V_f_ 40%)
Stiffness	[GPa]	17.12	21.54	72.12
CV	[%]	16	4	2
Failure strain	[%]	0.904	1.248	1.486
CV	[%]	25	6	3
Strength	[MPa]	148	262	1188
CV	[%]	17	5	4

**Table 3:** Pseudo-ductile ADFRCs tensile test results.

		CC_20-80		CG_20-80		CC_50-50		CG_40-60		
Composition		20% HMC 80% HSC		20% HSC 80% EG		50% HMC 50% HSC		40% HMC 60% EG		
		UD	QI	UD	QI	UD	QI	UD	QI	
Stiffness	[GPa]	85.3	28.4	53.2	17.8	118.8	40.0	77.5	26.9	
CV	[%]	6.1	5.2	1.3	3.3	4.2	2.8	5.1	7.0	
Yield strain	[%]	0.354	0.354	0.359	0.360	0.331	0.312	0.306	0.300	
CV	[%]	2.0	1.4	0.2	1.8	5.4	1.4	2.2	4.9	
Failure strain	[%]	1.420	1.323	1.870	1.646	1.248	1.086	1.496	1.206	
CV	[%]	0.4	0.3	7.4	2.3	5.6	4.3	1.0	4.4	
Strength	[MPa]	820	261	456	138	606	187	358	110	
CV	[%]	7.2	5.6	2.7	3.0	2.8	2.2	2.3	7.3	
Pseudo-ductile strain	[%]	0.456	0.404	1.013	0.868	0.738	0.618	1.034	0.797	
CV	[%]	22.6	2.3	13.0	4.4	10.3	5.8	3.1	5.4	
